# Effect of nusinersen on respiratory function in patients with spinal muscular atrophy: an 18-month single-center prospective study

**DOI:** 10.3389/fped.2026.1874241

**Published:** 2026-07-08

**Authors:** Tianjian Luo, Lingling Xu, Wen Tang, Liuyi Huang, Yijuan Li, Zhihui Yue

**Affiliations:** 1 Department of Pediatrics, The First Affiliated Hospital of Sun Yat-Sen University, Guangzhou, China; 2 Department of Pediatrics, Foshan Women and Children Hospital, Foshan, China

**Keywords:** active cough strength, neuromuscular disease, nusinersen, respiratory function, spinal muscular atrophy

## Abstract

**Background:**

Spinal muscular atrophy (SMA) is a severe neuromuscular disease characterized by respiratory muscle weakness, often leading to high mortality. Research on the effect of nusinersen on respiratory function in SMA patients remains scarce.

**Objective:**

To evaluate nusinersen's effect on respiratory function in SMA patients and provide a clinical basis for respiratory management.

**Methods:**

In this single-center prospective study, clinical data were collected from SMA patients in our pediatric department. Respiratory function, expressed as absolute values and percentages of predicted values (%pred), and active cough strength was evaluated at baseline and during follow-up after nusinersen treatment. Treatment effects were subsequently analyzed.

**Results:**

Among 59 SMA patients (39 Type II; 20 Type III), 40 completed baseline respiratory tests, of whom 67.5% had abnormal respiratory function. Type II patients had significantly lower VC %pred, FVC %pred, FEV1%pred, PEF %pred, FEF25%pred, and FEF50%pred than Type III patients (all *P* < 0.05). After 6 months, absolute value of FVC improved significantly in Type III (*P* < 0.05); by 18 months, absolute VC values in Type II patients, and absolute values and %pred of VC, FVC, FEF25, FEF50, and FEF75 in Type III increased significantly (all *P* < 0.05). Ineffective cough was observed in 76.3% of patients at baseline, and was more common in Type II patients (*P* = 0.001). Among patients who completed 18-month respiratory function follow-up (*n* = 23), the proportion with effective cough increased significantly from 39.1% (9/23) at baseline to 56.5% (13/23) after treatment (*P* < 0.05).

**Conclusion:**

Most SMA patients had impaired respiratory function, which was more severe in Type II than Type III patients. Nusinersen improved respiratory function and active cough strength, but could not fully reverse the progression of restrictive ventilatory dysfunction, especially in Type II.

## 1 Introduction

Spinal muscular atrophy (SMA) is an autosomal recessive neuromuscular disease caused by mutations in the survival motor neuron 1 (*SMN1*) gene on chromosome 5q13.2. These mutations result in a decrease in SMN protein, leading to neuronal degeneration and death, and progressive and symmetrical skeletal muscle atrophy. SMA poses a major threat to children's survival and health, and is the most common lethal genetic disorder in infants under 2 years of age (1, 2). SMA is classified into four Types (Types I-IV) based on the age of onset and the highest level of motor function achieved (3). As the disease progresses, lung expansion is significantly limited, pulmonary ventilation is severely insufficient, leading to carbon dioxide retention and hypoxia, and eventually progressing to respiratory failure. In addition, SMA patients have ineffective cough and decreased airway secretion clearance ability due to respiratory muscle weakness, resulting in sputum blockage, leading to dyspnea, atelectasis, and repeated respiratory tract infections. Therefore, respiratory failure is the most common cause of death in SMA patients (4).

Nusinersen, the first FDA-approved drug for SMA, is an antisense oligonucleotide that regulates *SMN2* pre-mRNA splicing, promoting the production of functional SMN protein. Multiple clinical trials and real-world studies have shown that after 12 or more months of nusinersen treatment, the motor function scores of patients with Type I-III SMA, such as the total score of the Children's Hospital of Philadelphia Infant Neuromuscular Disease Test (CHOP-INTEND) scale and the total score of the Hammersmith Functional Motor Scale Expanded Version (HFMSE), have improved, suggesting that the motor function of patients is improved to varying degrees (5–7).

However, few global studies have explored changes in respiratory function among SMA patients treated with nusinersen, and most of these studies are limited by small sample sizes and insufficient evaluation indicators (2, 8–12). Moreover, relevant real-world studies have reached conflicting conclusions: some reported long-term stability of respiratory function, whereas others noted a gradual decline in pulmonary ventilation indices (9, 13, 14) Given these unresolved issues, it remains unclear whether nusinersen can sustainably improve respiratory function and cough capacity in Type II and III SMA patients over an 18-month follow-up period.

Based on this, we conducted a single-center prospective study to systematically evaluate the long-term changes in respiratory function and active cough capacity in these patients.

## 2 Materials and methods

### 2.1 Study subjects

**Figure 1:**
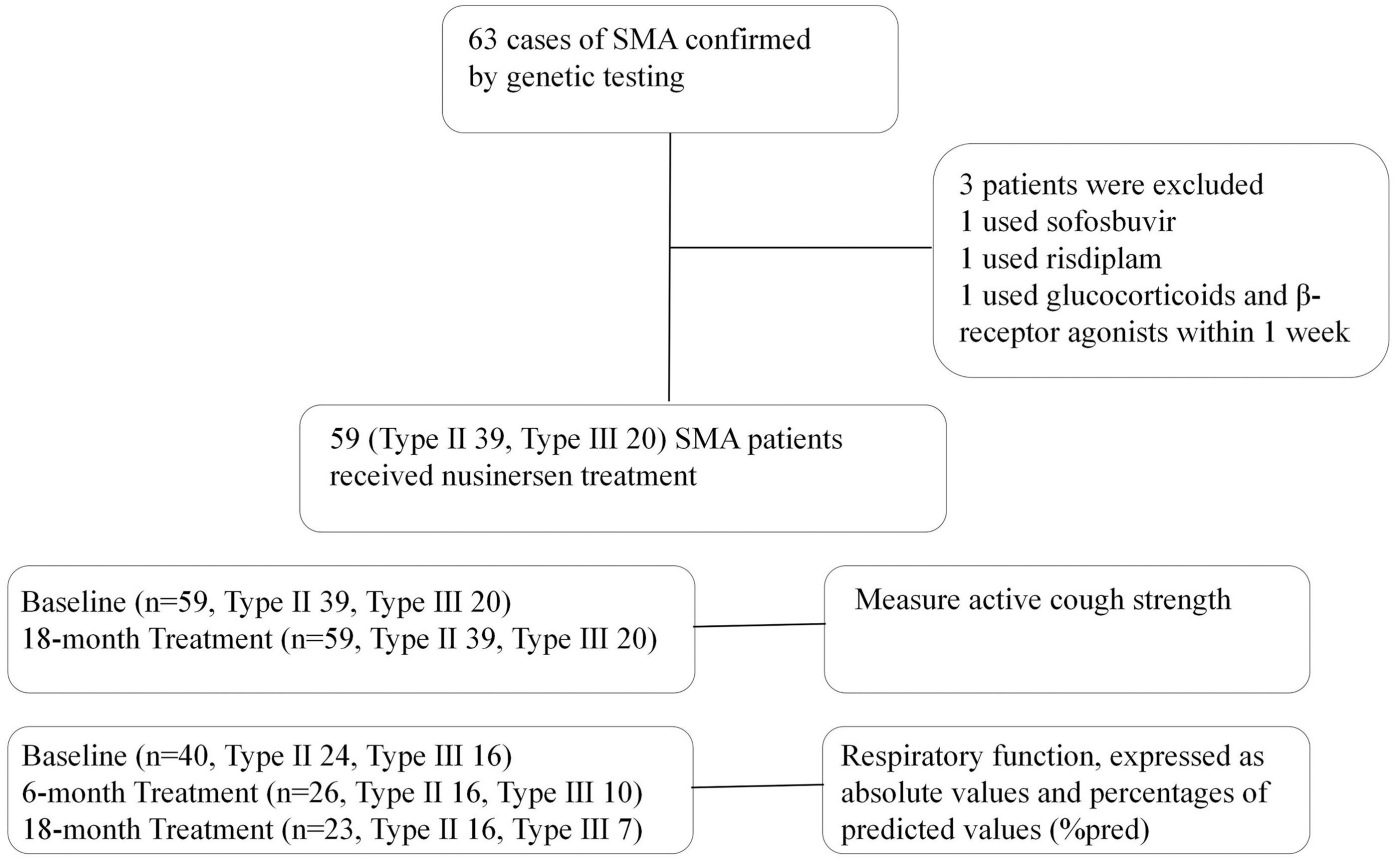
Patient flowchart. A total of 63 SMA patients were assessed; 3 were excluded. Finally, 59 patients were enrolled and received nusinersen. All 59 patients completed baseline and 18-month remote cough strength assessment. For on-site spirometry, 40 patients completed baseline tests, 26 completed 6-month follow-up, and 23 completed 18-month follow-up.

SMA patients admitted to the Department of Pediatrics at The First Affiliated Hospital of Sun Yat-sen University from January to October 2022 were included (Figure 1).

**Inclusion criteria:** (1) patients who had confirmed SMN1 gene mutations via genetic testing; (2) patients treated with nusinersen.

**Exclusion criteria:** (1) SMA patients who received other disease-modifying therapies (e.g., risdiplam) or investigational drugs (e.g., sofosbuvir used off-label); (2) SMA patients who had used glucocorticoids, *β*-receptor agonists (e.g., salbutamol), anticholinergic drugs (e.g., ipratropium bromide), leukotriene receptor blockers (e.g., montelukast sodium), or aminophylline within 1 week before enrollment; (3) SMA patients who were unable to complete pulmonary function tests due to severe disease or poor cooperation were excluded. We did not enroll Type I SMA patients in this study. According to recent literature, Type I SMA infants who received early standardized treatment can cooperate to finish pulmonary function tests. However, most Type I patients in our center had extremely severe physical conditions and could not complete effort-dependent spirometry, so they were not included in the present cohort.This study was approved by the Medical Ethics Committee of The First Affiliated Hospital of Sun Yat-sen University (approval number: Lun Shen [2021] 710) prior to patient enrollment. The patients and their guardians provided informed consent and signed the informed consent form.

Notably, active cough strength assessment could be conducted via standardized remote video assessment, so all 59 enrolled patients completed cough assessment at baseline and the 18-month follow-up. Spirometry required on-site physical examination, and thus only partial patients finished serial pulmonary function tests during follow-up. A detailed flowchart of patient enrollment, grouping and follow-up is shown in Figure 1.

### 2.2 Clinical data collection

(1) Demographic information: gender, age, weight, height (arm span); (2) SMA clinical classification, course of disease, respiratory symptoms and signs (presence or absence of inspiratory retractions, ineffective cough, scoliosis); (3) arterial partial pressure of oxygen, arterial partial pressure of carbon dioxide, and results of respiratory function tests. According to the age of onset and the maximum motor ability achieved, patients were classified into Type I–IV, in order of disease severity from severe to mild (15).

#### 2.2.1 Definition of normal respiratory function

Normal respiratory function was defined as all measured spirometry parameters meeting the following criteria: VC (%), FVC (%), FEV1 (%), PEF (%) ≥80%, and FEV1/FVC (% ≥92%.

#### 2.2.2 Definition of abnormal respiratory function

Abnormal respiratory function was defined as failure to meet all criteria for normal respiratory function, i.e., any one of the following: VC %pred <80%, FVC %pred <80%, FEV1%pred <80%, PEF %pred <80%, or FEV1/FVC %pred <92%.

#### 2.2.3 Definition of abnormal blood gas levels

“Abnormal blood gas levels” were defined as arterial partial pressure of oxygen <80 mmHg or arterial partial pressure of carbon dioxide >45 mmHg.

### 2.3 Nusinersen dosage and administration

Nusinersen was administered via intrathecal injection at a dose of 12 mg per administration. The first three doses were given at 2-week intervals, and the fourth dose was given 35 days after the third dose. Thereafter, nusinersen was administered every 4 months for maintenance treatment (16).

### 2.4 Respiratory function measures

Respiratory function was measured using a German Jaeger spirometer. Spirometry was conducted on-site by professional technicians. Spirometry was performed using a Jaeger device calibrated daily according to ATS/ERS (American Thoracic Society/European Respiratory Society) guidelines; the measured indices included VC (Vital Capacity), FVC (Forced Vital Capacity), FEV1 (Forced expiratory volume in 1 s), FEV1/FVC%, PEF (Peak expiration flow), FEF25 (Forced Expiratory Flow at 25% of vital capacity), FEF50, and FEF75, and the results were expressed as absolute values and percentages of predicted values (%pred). The %pred was calculated using the equations from Chinese pediatric pulmonary function guidelines (17). The indexes were evaluated according to internationally recognized standards: VC %pred, FVC %pred, FEV1%pred, and PEF %pred ≥80% were normal, 60%–79% were mildly decreased, 40%–59% were moderately decreased, and <40% were severely decreased; FEV1/FVC %pred ≥92% was normal, and FEF25%pred, FEF50%pred and FEF75%pred ≥65% were normal, 55%–64% were mildly decreased, 45%–54% were moderately decreased, and <45% were severely decreased. Restrictive ventilatory dysfunction was defined as FEV1/FVC %pred ≥92% and FVC %pred <80% (17); In this study, we also analyzed the reductions in FEF50 and FEF75. Such declines in patients with SMA are primarily attributable to reduced vital capacity and expiratory muscle weakness instead of structural small airway lesions.

### 2.5 Active cough strength measures

Active cough strength was graded into six levels by two pediatric pulmonologists, with assistance from the patients' caregivers, with higher levels indicating stronger cough strength. Levels 0–3 were defined as ineffective cough. Levels 4–5 were defined as effective cough (18) (Table 1). This 0–5 cough grading scale has been validated for pediatric SMA populations. All remote video assessments were independently performed by two senior pediatric pulmonologists. Inter-rater reliability was calculated, and the Cohen's Kappa coefficient was 0.87, indicating excellent consistency between raters.

**Table 1:** Grading scale for active cough strength (18).

Grading	Performance
Level 0	No cough on command
Level 1	Airflow sound can be heard in the trachea but no cough sound
Level 2	Very weak cough sound is heard
Level 3	Clear cough sound is heard
Level 4	Strong cough sound is heard
Level 5	Multiple strong coughs can be performed

Levels 0–3 were defined as ineffective cough; Levels 4–5 were defined as effective cough.

When patients could not return to the hospital for on-site assessment, cough strength evaluation was implemented via standardized remote follow-up.

### 2.6 Statistical analysis

Data were analyzed using SPSS 25.0 software. Normally distributed continuous data were described as mean ± standard deviation, and non-normally distributed continuous data were expressed as median (25th, 75th percentile). The independent-samples t-test was used to compare the respiratory function of Type II and Type III patients before treatment. The paired *t*-test or Wilcoxon signed-rank test was used to analyze the respiratory function between baseline and post-treatment in patients who completed follow-up, and the chi-square test was used to analyze the change in cough strength after treatment. The correlation between PEF %pred and ineffective cough was analyzed using Spearman's rank correlation coefficient in the 40 patients who completed baseline respiratory assessment. Individuals with missing data were excluded from the analysis, and a *P* value <0.05 was regarded as statistically significant.

For the analysis of treatment effects, changes in both absolute values and %pred were evaluated. Given the natural increase in lung volumes with growth in children, the analysis of %predvalues was critical for distinguishing drug effects from normal development.

## 3 Results

### 3.1 Clinical data

A total of 63 patients with suspected SMA were screened at first. Three patients were excluded, including two without confirmed SMN1 mutation and one refusing to join, leaving 59 patients enrolled in this prospective study (Figure 1).

All subjects finished baseline and 18-month active cough strength tests. For in-person spirometry, 40 patients completed baseline tests, 26 (16 Type II and 10 Type III patients) finished the 6-month follow-up, and 23 (16 Type II and 7 Type III patients) completed the 18-month follow-up. Of the 40 patients with baseline spirometry, 36 dropped out within 18 months: 19 withdrew before month 6, and another 17 were lost to follow-up between month 6 and month 18, leading to a 42.5%(17/40) dropout rate for the spirometry group.

Patients mainly dropped out because of long travel distance for hospital visits, poor child cooperation during lung function tests, or switching to local hospitals for regular treatment. The 23 patients who finished full 18-month spirometry had better baseline cough function, with a baseline effective cough rate of 39.1%, almost double the overall cohort's 23.7%. It suggests the retained patients were in better physical condition, thus introducing survivorship bias and selection bias in the longitudinal analysis. Age, gender, disease course and key baseline lung function indicators showed no obvious differences between dropout and retained patients (all *P* > 0.05).

Among the 59 SMA patients, 36 (61.0%) had scoliosis. The scoliosis rate was 64.1% (25/39) in Type II patients and 55.0% (11/20) in Type III patients. Statistical analysis found no significant inter-group difference in scoliosis incidence (*P* = 0.497).

#### 3.1.2 Use of respiratory assistive devices

For respiratory assistive devices, none of the 59 patients used cough assist machines during follow-up. Only two patients needed temporary non-invasive ventilation (NIV) when they had lung infections, and they stopped NIV once infections cleared; no patients received long-term regular NIV. In subtype analysis, 2 out of 39 Type II patients used short-term NIV (5.1%), while all 20 Type III patients did not use any respiratory aids (0%). By motor function, short-term NIV rates were 7.1% (1/14) for non-sitters, 3.6% (1/28) for sitters-nonwalkers and 0% (0/17) for walkers. No patients started long-term respiratory assistive devices at baseline or the 18-month follow-up (Table 2).

**Table 2:** Usage of respiratory assistive devices in different subgroups at baseline and 18-month follow-up.

Subgroup	Total cases	Patients using NIV (n, %)	Patients using cough assist device (n, %)	Notes
SMA subtype				
Type II	39	2 (5.1)	0 (0.0)	Short-term use for pneumonia
Type III	20	0 (0.0)	0 (0.0)	No device used
Total	59	2 (3.4)	0 (0.0)	
Motor function stratification				
Non-sitter	14	1 (7.1)	0 (0.0)	Short-term use for pneumonia
Sitter-non-walker	28	1 (3.6)	0 (0.0)	Short-term use for pneumonia
Walker	17	0 (0.0)	0 (0.0)	No device used

NIV, non-invasive ventilation.

#### 3.1.3 Standardized respiratory physiotherapy

All enrolled patients received the same respiratory physiotherapy for 18 months, including postural drainage and chest percussion. Each patient took this therapy twice a day for 15 min each time, with fixed frequency, length and operation steps throughout the study. All subjects followed the same rehabilitation plan. This unified treatment removed the interference of different physiotherapy schemes when evaluating respiratory function and cough strength.

### 3.2 Respiratory function before nusinersen treatment

Forty patients finished lung function tests before treatment, including 24 Type II (60.0%) and 16 Type III (40.0%) patients. Twenty-seven patients (67.5%) had abnormal lung function, and nine had reduced FEV1/FVC%. Most patients kept normal FEV1/FVC ratios, a typical sign of neuromuscular restrictive ventilatory dysfunction. Respiratory muscle weakness equally lowers lung volume and expiratory flow instead of damaging airways themselves. Twenty-nine patients had low PEF %pred, 26 had low VC %pred, 26 had low FVC %pred, 20 had low FEV1%pred, and 17 were diagnosed with restrictive ventilatory dysfunction. Thirteen patients had reduced FEF50 and FEF75, mainly due to smaller lung volume and weak expiratory muscles in SMA.

Among the 29 patients with reduced PEF %pred, 23 had ineffective cough, and PEF %pred was negatively correlated with poor cough (*r* = −0.452, *P* = 0.007). Before treatment, Type II patients had much lower VC %pred, FVC %pred, FEV1%pred, PEF %pred, FEF25%pred and FEF50%pred than Type III patients (all *P* < 0.05). FEV1/FVC %pred and FEF75%pred showed no obvious differences between two subtypes (both *P* > 0.05) (Table 3).

**Table 3:** Clinical characteristics of 59 SMA patients before treatment.

General clinical data (*n* = 40)				
Variables	Total	Type II	Type III	*P*
No of cases	59	39	20	
Sex (n, %)				0.625
Male	33 (55.9)	21 (53.8)	12 (60.0)	
Female	26 (44.1)	18 (46.2)	8 (40.0)	
Age of treatment initiation (y)	8.0 (4.8, 10.0)	7.0 (4.0, 9.0)	9.0 (6.0, 12.8)	0.053
Disease duration (y)	6.0 (3.5, 7.4)	6.0 (3.5, 7.4)	7.0 (4.1, 9.0)	0.088
Active cough strength (n, %)				
No of cases	59	39	20	
Ineffective cough	45 (76.3)	37 (94.9)	8 (40.0)	<0.001
Effective cough	14 (23.7)	2 (5.1)	12 (60.0)	<0.001
Level 0	8 (13.6)	8 (20.5)	0 (0.00)	0.042
Level 1	13 (22.0)	10 (25.6)	3 (15.0)	0.511
Level 2	20 (33.9)	17 (43.6)	3 (15.0)	0.042
Level 3	4 (6.78)	2 (5.1)	2 (13.3)	0.598
Level 4	7 (11.8)	2 (5.1)	5 (25.0)	0.038
Level 5	7 (11.8)	0 (0.0)	7 (35.0)	＜0.001
Scoliosis (n, %)	36 (61.0)	25 (69.4)	11 (30.6)	0.497
Respiratory Function (*n* = 40)				
No of cases	40	24	16	
Normal respiratory Function (n, %)	13 (32.5)	4 (16.7)	9 (56.3)	0.015
Abnormal respiratory Function (n, %)	27 (67.5)	20 (83.3)	7 (43.8)	0.015
Restrictive ventilatory dysfunction (n, %)	17 (42.5)	15 (62.5)	2 (12.5)	0.003
Reduced FEF50 and FEF75 (n, %)	13 (32.5)	12 (50.0)	1 (6.3)	0.005
FEV1/FVC %pred Decreased (n, %)	9 (22.5)	3 (12.5)	6 (37.5)	0.120
PEF %pred Decreased (n, %)	29 (72.5)	20 (83.3)	9 (56.3)	0.080
VC %pred Decreased (n, %)	26 (65.0)	21 (87.5)	5 (31.3)	＜0.001
FVC %pred Decreased (n, %)	26 (65.0)	20 (83.3)	6 (37.5)	0.006
FEV1%pred Decreased (n, %)	20 (50.0)	16 (66.7)	4 (25.0)	0.023
VC %pred	72.5 (46.3, 96.4)	55.4 (39.7, 75.9)	94.4 (75.2, 105.5)	0.001
FVC %pred	73.5 (48.2, 99.4)	51.8 (38.2, 77.4)	95.4 (75.7, 110.3)	0.001
FEV1%pred	78.8 (46.7, 104.0)	60.4 ± 32.9	93.9 ± 24.2	0.004
FEV1/FVC %pred	104.7 (96.6, 108.6)	105.1 (97.7, 108.6)	103.9 (87.1, 110.5)	0.689
PEF %pred	61.3 ± 26.1	52.8 ± 22.7	75.4 ± 25.3	0.032
FEF25%pred	66.5 ± 28.4	57.0 ± 25.0	80.7 ± 27.8	0.008
FEF50%pred	67.7 ± 36.2	58.5 ± 34.2	81.5 ± 35.9	0.049
FEF75%pred	55.0 (28.4, 100.2)	51.9 (11.3, 81.8)	84.1 (47.1, 103.2)	0.077

Levels 0–3 were defined as ineffective cough; Levels 4–5 were defined as effective cough; Respiratory function parameters were analyzed in 40 patients (total *n* = 40; Type II, *n* = 24; Type III, *n* = 16).PEF, Peak expiration flow; VC, Vital capacity; FVC, Forced Vital Capacity; FEV1, Forced expiratory volume in 1 s; PEF, Peak expiration flow; Restrictive ventilatory dysfunction: FEV1/FVC %pred ≥92% and FVC %pred <80%; reduced FEF50 and FEF75: FEV1/FVC %pred ≥92%, with both FEF50%pred and FEF75%pred <65%.

Further subgroup analysis by motor status (non-sitter, sitter-non-walker, walker) found baseline VC %pred, FVC %pred and expiratory flow indicators dropped as motor function got worse. Non-sitter patients suffered the worst restrictive ventilatory impairment.

### 3.3 Respiratory function variations after nusinersen treatment

For respiratory function analysis, only patients who completed spirometry were included: 40 at baseline, 26 at 6 months, and 23 at 18 months (Figure 1). Tables 4, 5 summarized the serial pulmonary function data of patients who completed spirometry at both 6-month and 18-month follow-up.

**Table 4:** Absolute respiratory function values of 16 type II SMA patients (baseline, 6-month treatment, 18-month treatment).

Variables	0 (*n* = 16)	6M (*n* = 16)	18M (*n* = 16)
VC (L)	0.9 ± 0.4	1.1 ± 0.4	1.1 ± 0.6*
FVC (L)	0.8 (0.5, 1.4)	1.1 (0.7, 1.5)	1.3 (0.6, 1.6)
FEV1 (L/s)	0.9 (0.5, 1.3)	0.9 (0.5, 1.2)	1.1 (0.5, 1.4)
FEV1/FVC %	90.9 (82.8, 93.4)	82.9 (55.8, 87.4)	84.6 (79.0, 88.7)
PEF (L/s)	1.7 (1.3, 2.5)	1.7 ± 0.7	2.0 (1.3, 2.7)
FEF25 (L/s)	1.8 (1.3, 2.4)	1.8 (0.8, 2.2)	1.6 (1.1, 2.3)
FEF50 (L/s)	1.3 ± 0.7	1.1 (0.6, 1.7)	1.1 ± 0.6
FEF75 (L/s)	0.6 ± 0.5	0.5 ± 0.3	0.5 ± 0.3
VC %pred	54.3 ± 20.0	56.7 ± 18.5	46.1 ± 18.5*
FVC %pred	53.9 ± 21.1	59.1 ± 19.1	53.5 ± 22.8
FEV1%pred	53.7 ± 23.2	56.0 ± 19.5	46.8 ± 23.2
FEV1/FVC %pred	105.6 (97.2, 108.1)	101.3 (97.2, 105.2)	93.7 (86.5, 103.7)
PEF %pred	50.9 ± 20.7	50.4 ± 20.1	44.5 ± 17.8*
FEF25%pred	54.5 (36.1, 75.8)	52.9 (34.8, 64.5)	44.2 ± 20.2*
FEF50%pred	50.9 (30.4, 83.3)	56.9 (37.6, 77.7)	42.6 ± 22.3*
FEF75%pred	42.6 (10.8, 83.4)	43.6 (23.8, 67.6)	36.2 (19.4, 48.2)

0, Baseline; 6M, 6-month Treatment; 18M, 18-month Treatment; VC, Vital capacity; FVC, Forced Vital Capacity; FEV1, Forced expiratory volume in 1 s; FEV1/FVC %, Measured FEV1/FVC ratio; FEV1/FVC (%), FEV1/FVC percentages of predicted values; PEF, Peak expiration flow; FEF25, Forced Expiratory Flow at 25% of vital capacity; *P*-value shows each group's statistical comparison with the Baseline group.

* *P* < 0.05 vs. baseline.

This table included 16 Type II SMA patients who completed spirometry at baseline, 6-month and 18-month follow-up, selected from the 39 overall Type II patients.

**Table 5:** Absolute respiratory function values in patients with SMA type III (baseline, 6-month treatment, 18-month treatment).

Variables	0 (*n* = 10)	6M (*n* = 10)	0 (*n* = 7)	18M (*n* = 7)
VC (L)	2.3 ± 1.0	2.6 ± 1.2	2.3 ± 1.0	3.0 ± 1.1*
FVC (L)	2.4 ± 1.0	2.6 ± 1.2*	2.4 ± 1.0	3.0 ± 1.1*
FEV1 (L/s)	2.0 ± 0.9	2.2 ± 0.9	2.2 ± 0.9	2.7 ± 1.0
FEV1/FVC %	80.0 ± 14.0	86.4 ± 11.9	80.7 ± 14.0	90.5 ± 5.4
PEF (L/s)	3.1 (2.0, 5.0)	3.5 (3.0, 4.5)	3.8 (2.6, 5.5)	4.2 (3.5, 7.1)
FEF25 (L/s)	2.8 (2.3, 4.6)	2.9 (2.2, 4.2)	2.8 ± 1.3	4.0 ± 1.3*
FEF50 (L/s)	2.2 (1.7, 3.3)	1.6 (1.5, 3.5)	2.0 ± 1.1	3.0 ± 1.2*
FEF75 (L/s)	1.1 (0.8, 1.7)	0.6 (0.5, 1.8)	1.0 ± 0.5	2.0 ± 0.7*
VC %pred	93.0 (75.0, 97.9)	81.2 (73.0, 89.6)	93.0 (75.0, 97.9)	86.5 (81.3, 88.7)*
FVC %pred	95.4 (79.7, 101.4)	82.2 (73.6, 91.0)*	95.4 (79.7, 101.4)	85.6 (81.9, 97.1)*
FEV1%pred	93.4 (82.9, 112.3)	78.8 (67.5, 100.0)	93.4 (82.9, 112.3)	99.4 (85.4, 104.7)
FEV1/FVC %pred	107.0 (88.4, 111.2)	105.0 (93.1, 113.5)	107.0 (88.4, 111.2)	107.9 (103.6, 109.1)
PEF %pred	76.3 (72.7, 93.7)	71.4 (67.3, 86.2)	76.3 (72.7, 93.7)	84.9 (81.5, 86.8)
FEF25%pred	87.2 (81.2, 105.2)	73.5 (70.3, 94.3)	87.2 (81.2, 105.2)	89.4 (69.4, 94.9)
FEF50%pred	100.5 (90.3, 109.6)	53.8 (53.4, 92.4)	100.5 (90.3, 109.6)	80.2 (50.0, 93.6)
FEF75%pred	94.8 (51.5, 102.7)	41.2 (36.7, 107.3)	94.8 (51.5, 102.7)	84.0 (56.0, 118.5)

0, baseline; 6M, 6-month Treatment; 18M, 18-month Treatment; VC, Vital capacity; FVC, Forced Vital Capacity; FEV1, Forced expiratory volume in 1 s; FEV1/FVC %, Measured FEV1/FVC ratio; FEV1/FVC (%), FEV1/FVC percentages of predicted values; PEF, Peak expiration flow; FEF25, Forced Expiratory Flow at 25% of vital capacity; *P*-value shows each group's statistical comparison with the baseline group;.

* *P* < 0.05 vs. baseline.

A total of 10 Type III patients completed spirometry at baseline and 6-month follow-up, and 7 of them continued to finish the 18-month spirometry test. All subjects were selected from the total 20 Type III patients enrolled in the study.

After 6 months of treatment, respiratory function indices in Type II patients showed no significant change (*P* > 0.05) in both absolute values and %pred; Absolute FVC values in Type III patients increased significantly (*P* < 0.05), while FVC %pred decreased significantly (*P* < 0.05) compared to baseline.

**Figure 2:**
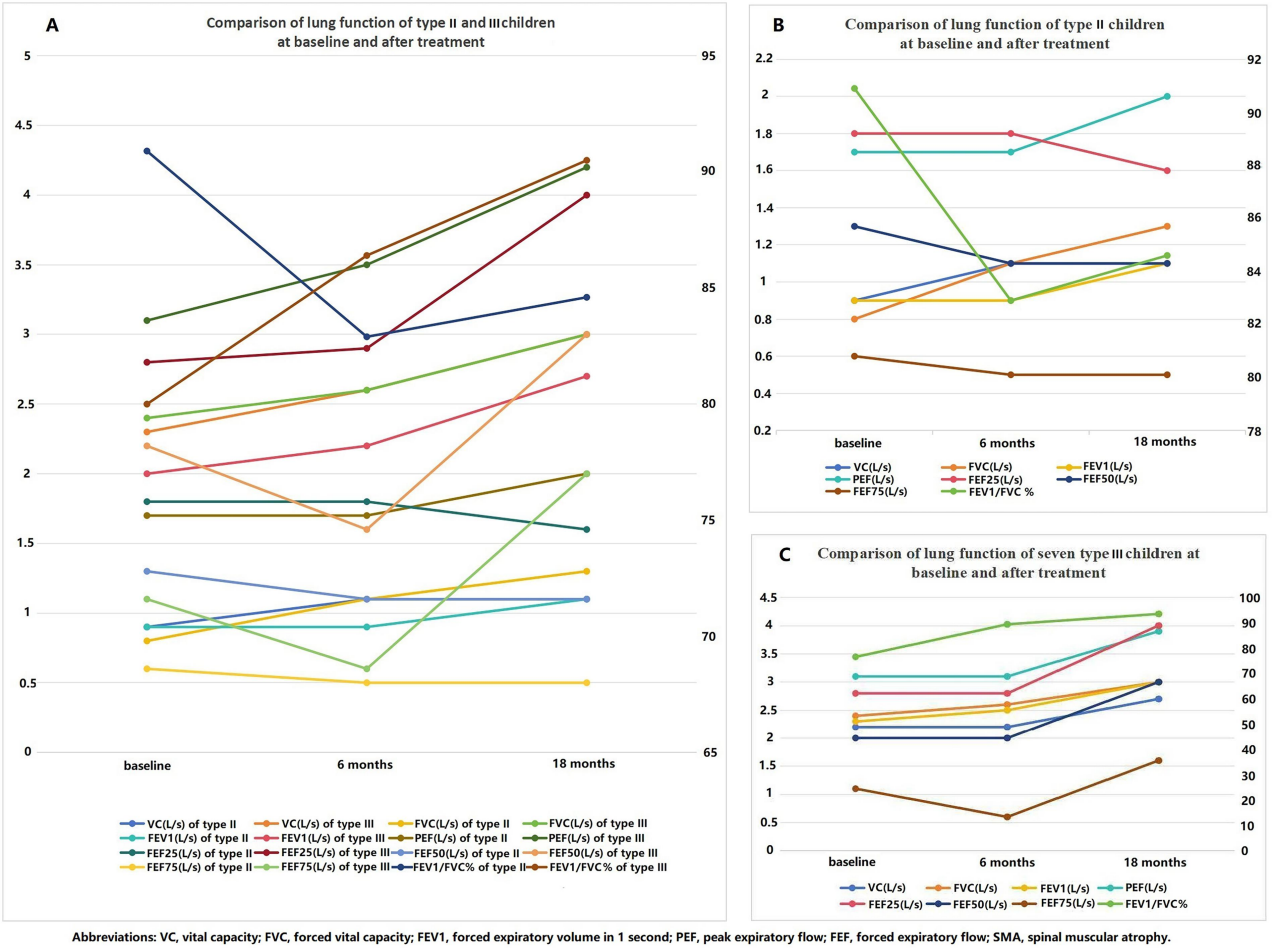
Absolute respiratory function values of SMA patients (baseline, 6-month treatment, 18-month treatment). **(A)** Type II and III comparison; **(B)** Type II alone; **(C)** Type III alone; 0, Baseline; 6M, 6-month Treatment; 18M, 18-month Treatment; VC, Vital capacity; FVC, Forced Vital Capacity; FEV1, Forced expiratory volume in 1 s; PEF, Peak expiration flow; FEF25, Forced Expiratory Flow at 25% of vital capacity; *P*-value shows each group's statistical comparison with the Baseline group. *indicates *P* < 0.05; ** indicates *P* < 0.01, comparing to Baseline group; # indicates *P* < 0.05.

**Figure 3:**
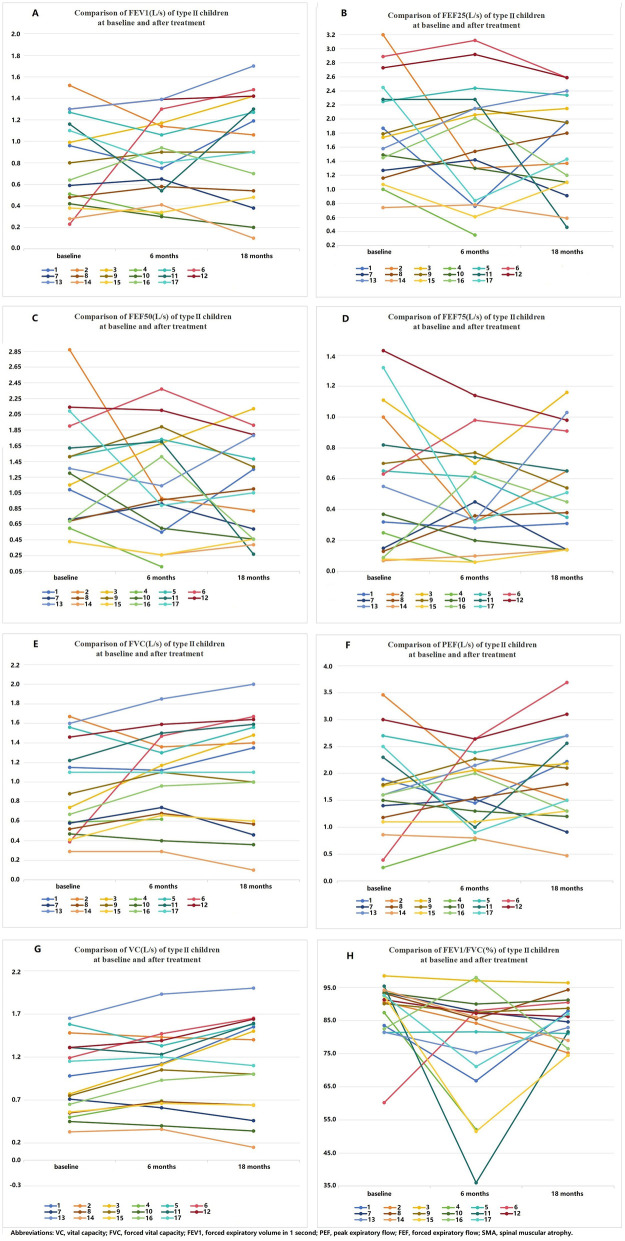
Absolute respiratory function values of type II SMA patients (baseline, 6-month treatment, 18-month treatment): FEV1 (L/s) **(A)**; FEF25 (L/s) **(B)**; FEF50 (L/s) **(C)**; FEF75 (L/s) **(D)**; FVC (L/s) **(E)**; PEF (L/s) **(F)**; VC (L/s) **(G)**; FEV1/FVC (%) **(H)**.

**Figure 4:**
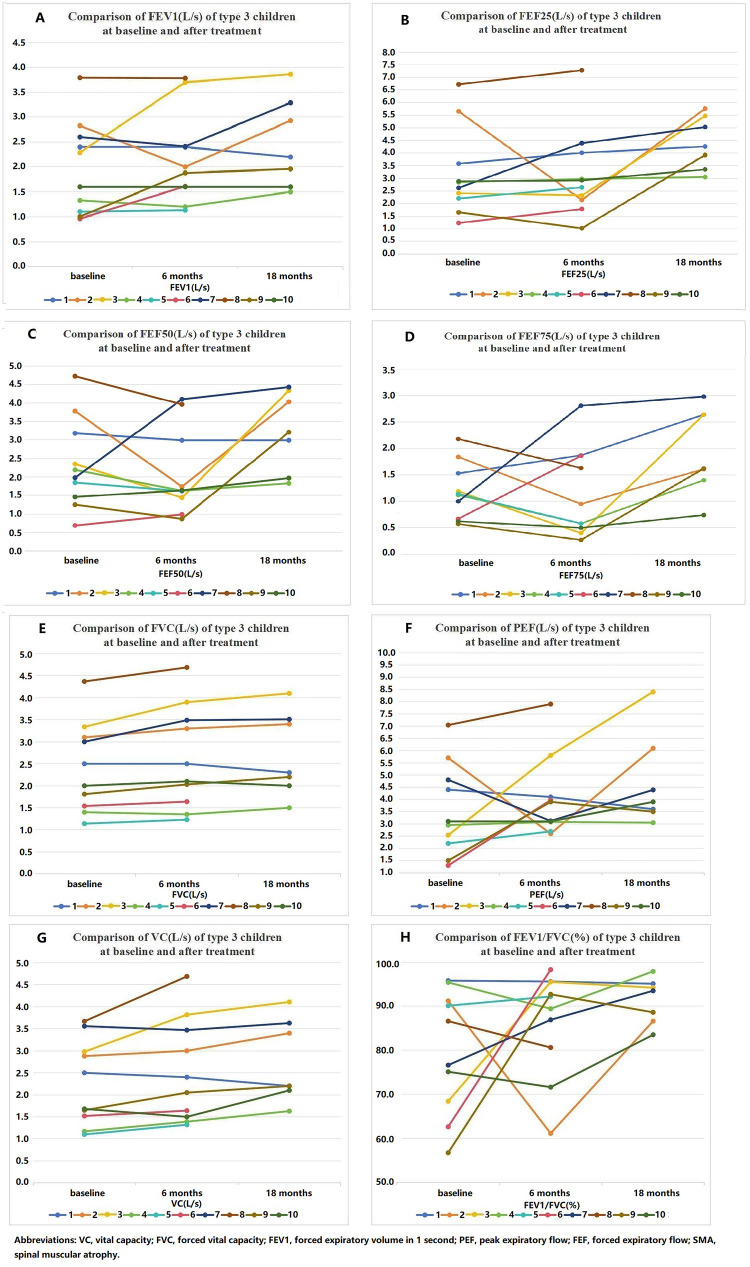
Absolute respiratory function values of type III SMA patients (baseline, 6-month treatment, 18-month treatment): FEV1 (L/s) **(A)**; FEF25 (L/s) **(B)**; FEF50 (L/s) **(C)**; FEF75 (L/s) **(D)**; FVC (L/s) **(E)**; PEF (L/s) **(F)**; VC (L/s) **(G)**; FEV1/FVC (%) **(H)**.

After 18 months of treatment, the absolute VC values of Type II patients increased significantly (*P* = 0.045). Driven by physical growth, the growth of lung volume failed to reach the standard of age-matched healthy children, so VC %pred decreased significantly (*P* = 0.039) compared with baseline. In Type III patients, absolute values of VC, FVC, FEF25, FEF50 and FEF75 increased significantly. Similarly, although the absolute pulmonary volume rose with body development, the increment was inferior to the expected level of healthy peers, leading to a significant reduction in %pred of VC and FVC (*P* < 0.05).All results for absolute values and %pred are presented in Tables 4, 5 (Figures 2–4).

This changing trend was consistent with the natural growth patterns of children and the progressive restrictive ventilatory dysfunction caused by SMA. Stratified analysis based on motor functional status showed that patients in sitter-non-walker and walker subgroups had more significant improvements in absolute lung volume after treatment, while respiratory indicators changed slightly in the non-sitter subgroup.

### 3.4 Active cough strength variations subsequent to nusinersen treatment

All 59 patients received active cough strength tests at baseline and the 18-month follow-up through remote assessment. Cough strength changes were further analyzed in 23 patients who finished the complete 18-month spirometry follow-up (Table 1, Table 6). No patients had respiratory distress or abnormal blood gas results at baseline. Overall, 76.3% (45/59) of patients had ineffective cough and 23.7% (14/59) had effective cough at baseline (Table 3). Type II patients had a significantly lower effective cough rate than Type III patients (5.1% vs. 60.0%, *P* < 0.001), and level 2 cough was more prevalent in Type II patients (43.6% vs. 15.0%, *P* = 0.042).

**Table 6:** Active cough strength in 59 SMA patients after 18 months of treatment.

Variables	Total	Clinical Type		*P*
		Type II	Type III	
No of cases	59	39	20	
Active cough strength, *n* (%)				
Ineffective cough	43 (72.9)	35 (89.7)	8 (40.0)	<0.001
Effective cough	16 (27.1)	4 (10.3)	12 (60.0)	<0.001
Level 0	3 (5.1)	3 (7.7)	0 (0.0)	0.544
Level 1	4 (6.8)	3 (7.7)	1 (5.0)	1.000
Level 2	17 (28.8)	15 (38.5)	2 (10.0)	0.048
Level 3	19 (32.2)	14 (35.9)	5 (25.0)	0.580
Level 4	9 (15.3)	4 (10.3)	5 (25.0)	0.249
Level 5	7 (11.9)	0 (0.0)	7 (35.0)	<0.001

Levels 0–3 were defined as ineffective cough; Levels 4–5 were defined as effective cough.

After 18 months of nusinersen treatment, cough strength was assessed by standardized remote evaluation with unified criteria to guarantee detection reliability. The overall effective cough rate rose from 23.7% to 27.1%, without significant statistical difference (*P* > 0.05). However, among the 23 patients who completed full 18-month respiratory follow-up, active cough strength was significantly improved, and the effective cough rate increased from 39.1% (9/23) to 56.5% (13/23) (*P* = 0.043). Notably, this beneficial result was only found in this relatively healthy subgroup. Considering the high dropout rate and inherent survivorship bias, this improvement cannot be extended to all Type II and Type III SMA patients treated with nusinersen. Subgroup analysis showed that the effective cough rate increased from 12.5% (2/16) to 25.0% (4/16) in 16 Type II patients, and from 57.1% (4/7) to 71.4% (5/7) in 7 Type III patients, and no significant differences were found in both subgroups.

### 3.5 Subgroup analysis by motor function

According to the current SMA functional milestone classification criteria, all 59 enrolled patients were divided into three subgroups based on baseline motor function, including the non-sitter group (*n* = 14), sitter-non-walker group (*n* = 28) and walker group (*n* = 17). There were 39 Type II patients (14 non-sitters, 23 sitter-non-walkers and 2 walkers) and 20 Type III patients (5 sitter-non-walkers and 15 walkers) in total. After 18 months of nusinersen treatment, the average active cough strength score increased significantly in all three subgroups (all *P* < 0.05). Specifically, the mean score rose from 1.07 (15/14) to 1.93 (27/14) in the non-sitter subgroup, from 1.89 (53/28) to 2.57 (72/28) in the sitter-non-walker subgroup, and from 3.53 (60/17) to 3.94 (67/17) in the walker subgroup (Table 7). Due to the high patient dropout rate and incomplete follow-up data, further subgroup analysis on pulmonary function as well as correlation analysis between motor function improvement and respiratory function improvement could not be conducted.

**Table 7:** Active cough strength scores by motor function subgroup and SMA type (baseline, 18-month treatment).

Subgroup	Type	0 (total/patients, mean)	18M (total/patients, mean)
Non-sitter	II (*n* = 14)	15/14 (1.07)	27/14 (1.93)*
Sitter-non-walker	II (*n* = 23)	39/23 (1.70)	58/23 (2.52)
	III (*n* = 5)	14/5 (2.80)	14/5 (2.80)
	Total (*n* = 28)	53/28 (1.89)	72/28 (2.57)*
Walker	II (*n* = 2)	4/2 (2.00)	6/2 (3.00)
	III (*n* = 15)	56/15 (3.73)	61/15 (4.07)
	Total (*n* = 17)	60/17 (3.53)	67/17 (3.94)*

0, Baseline; 6M, 6-month Treatment; 18M, 18-month Treatment.

* *P* < 0.05 vs. baseline.

## 4 Discussion

SMA is a severe neuromuscular disorder featuring skeletal muscle atrophy and weakness with multi-system involvement, and respiratory failure is a common cause of death (4). Current guidelines stress multidisciplinary care and regular respiratory function assessment for SMA patients (2). Previous studies report 39% of Type II and 9% of Type III patients need noninvasive ventilation at median ages of 5.0 and 15.1 years respectively (19). Among the 59 baseline patients in our study, 76.3% had ineffective cough at baseline, and Type II patients showed much worse pulmonary function and active cough strength than Type III ones. For the 23 patients finishing the 18-month respiratory follow-up, active cough strength improved greatly, with the rate of effective cough rising from 39.1% (9/23) to 56.5% (13/23). Patients who could sit or walk gained more benefits across all 59 subjects.

Effective cough relies on coordination of multiple muscles. In SMA patients, weak intercostal muscles fail to build sufficient intrapulmonary pressure and airflow velocity, leading to ineffective cough. Clinically, patients cannot cough well when peak cough flow (PCF) <160 L/min or maximum expiratory pressure (MEP) <45 cmH₂O (20, 21). Previous studies reported that MEP and PCF declined 12 months after symptom onset in patients with Type II and Type III SMA (22). Our analysis of 40 patients with complete baseline respiratory data revealed that reduced PEF %pred was strongly correlated with ineffective cough (*r* = −0.452, *P* = 0.007), indicating cough strength can roughly reflect overall respiratory function in clinical practice.

Consistent with prior reports (23, 24), our 40 baseline subjects showed markedly poorer pretreatment respiratory function in Type II than Type III SMA, which supports subtype-specific respiratory management. Two domestic cohorts (Xu et al.: 28 patients, 22 Type II/ 6 Type III (24); Jiang et al.: 29 patients, 18 Type II/11 Type III (23)) both identified decreased FEV1%pred with normal FEV1/FVC, implying shared airway features across SMA subtypes worthy of further exploration. Our data also match several small-scale studies (Banda et al., 16 Type II (7); Mao et al., 15 patients (2); Zhu et al., 19 patients (12)). Although we enrolled 59 patients at baseline, longitudinal efficacy analysis only included 26 patients at Month 6 and 23 at Month 18; still, this long-term dataset supports the respiratory benefits of nusinersen.

Notably, respiratory improvements in followed patients were partially independent of motor gains. We did not systematically collect HFMSE and other motor scale scores to perform full correlation analysis, but clinical observation showed some patients had obvious cough strength improvement without visible motor function changes. This suggests nusinersen may directly or indirectly protect respiratory muscles including the diaphragm and intercostal muscles (9).During disease progression, the diaphragm is relatively preserved while intercostal weakness mainly impairs cough reflex and chest expansion (25, 26).The prominent cough improvement in our cohort indicates nusinersen acts differently on motor neurons controlling respiratory muscles, boosting cough function regardless of overall somatic motor performance. Preclinical and clinical evidence backs this hypothesis: multiple studies confirm nusinersen elevates SMN protein in respiratory neurons and slows degeneration of thoracic motor neurons (8, 27).

Such improvements carry critical clinical value. Respiratory function largely determines quality of life for SMA patients. Given obvious survivorship bias in this longitudinal study, we cannot conclude uniform respiratory benefits for all SMA patients. Stronger effective cough helps clear airway secretions and lowers risks of pulmonary infection and hospitalization. The significant cough strength gains among our 23 fully followed patients allow better daily respiratory management, such as less suctioning and reduced ventilator reliance, further improving exercise tolerance, sleep quality and lowering caregiver burden (20, 28).

Only a small number of patients used short-term NIV during infection, and none used cough assist devices. Device use did not alter assessment timing or differ between subtypes, removing mechanical interference and ensuring accurate evaluation of nusinersen's efficacy.

Thirty-six out of 59 enrolled patients (61.0%) developed scoliosis, with incidence of 69.4% in Type II and 30.6% in Type III SMA. Progressive muscle weakness in SMA leads to scoliosis, which further limits chest wall expansion and thoracic compliance and exacerbates restrictive ventilatory dysfunction. More severe muscle atrophy and scoliosis in Type II patients account for their poorer respiratory function relative to Type III patients. Though we did not stratify patients by scoliosis severity, scoliosis is widely acknowledged to aggravate ventilation restriction. Our 18-month longitudinal data prove high scoliosis prevalence is a key factor driving gradual declines in pulmonary function %pred, especially for higher-risk Type II patients. Beyond mechanical compression, genetic factors may also contribute to this trend.

Respiratory impairment severity varied sharply by subtype, mainly due to different respiratory muscle strength. Type II SMA usually carries 3 SMN2 copies while Type III often has 3–4 copies; SMN2 copy number inversely correlates with disease severity (29–32). However, SMN2 copy number testing was not performed for patients in our cohort.

In SMA, skeletal muscles undergo progressive atrophy and external intercostal muscle weakness worsens gradually; natural history studies show predicted respiratory function values decline steadily with age (3, 33, 34). Although nusinersen has the aforementioned benefits, the 23 patients who completed the 18-month follow-up still presented reduced %pred of pulmonary indices including VC %pred after treatment, and this contrast of increased absolute lung volumes but decreased %pred is mainly caused by the long follow-up period since continuous thoracic growth in children naturally lifts absolute lung volume. Nusinersen enhances respiratory muscle strength and absolute pulmonary measurements, yet such improvements fail to keep up with lung development in healthy peers of the same age and thus lead to lower %pred. Taken together, these results reveal that nusinersen can increase absolute respiratory indicators and cough strength but cannot fully block progressive restrictive ventilatory impairment, especially in children with SMA Type II.

## 5 Limitations

This study has several limitations. First, it was a single-center prospective observational study without an untreated control group, which may underestimate the therapeutic efficacy of nusinersen. The participant dropout rate was about 42.5% over the 18-month follow-up, causing obvious survivorship and selection bias: patients finishing full follow-up had better baseline cough function and physical status than the whole cohort, so the observed cough strength improvements cannot represent all patients. Participants dropped out mainly because some families chose local treatment for easier drug access, while others refused respiratory tests due to insufficient disease knowledge. Most patients lacked SMN2 copy number data, limiting analyses linking clinical features and genetic phenotypes. Besides, the follow-up overlapped with the COVID-19 pandemic; strict pandemic control restricted hospital visits, and we failed to collect 12-month pulmonary function data for interim analysis. Motor function scales including HFMSE were not routinely assessed, so we could not analyze correlations between motor and respiratory improvements. Though single-center design introduced selection bias, strict inclusion criteria reduced confounders. Future studies should expand sample size and prolong follow-up to acquire more reliable data on post-treatment respiratory changes.

## 6 Conclusion

Nusinersen improved respiratory function and cough strength in patients finishing the 18-month follow-up, but no significant cough improvement was seen in the whole cohort. Patients with better motor function gained greater benefits when stratified by motor status (non-sitter, sitter-non-walker, walker). Still, the drug cannot fully reverse restrictive ventilatory dysfunction vs. healthy peers, especially for SMA Type II patients with worse and less reversible respiratory damage than Type III. These results provide useful clinical evidence for SMA respiratory care and further study.

## Data Availability

The original contributions presented in the study are included in the article/Supplementary Material, further inquiries can be directed to the corresponding author/s.
